# Supplementary material for: In vivo cell biological screening identifies an endocytic capture mechanism for T-tubule formation
Source: Nat Commun. 2020 Jul 24;11:3711. doi: 10.1038/s41467-020-17486-w (PMC7381618; doi:10.1038/s41467-020-17486-w)
Supplement: Supplementary file 1 — Supplementary Information [file 41467_2020_17486_MOESM1_ESM.pdf]

SUPPLEMENTARY INFORMATION

In vivo cell biological screening identifies an endocytic capture mechanism for T-tubule formation

Hall *et al*.

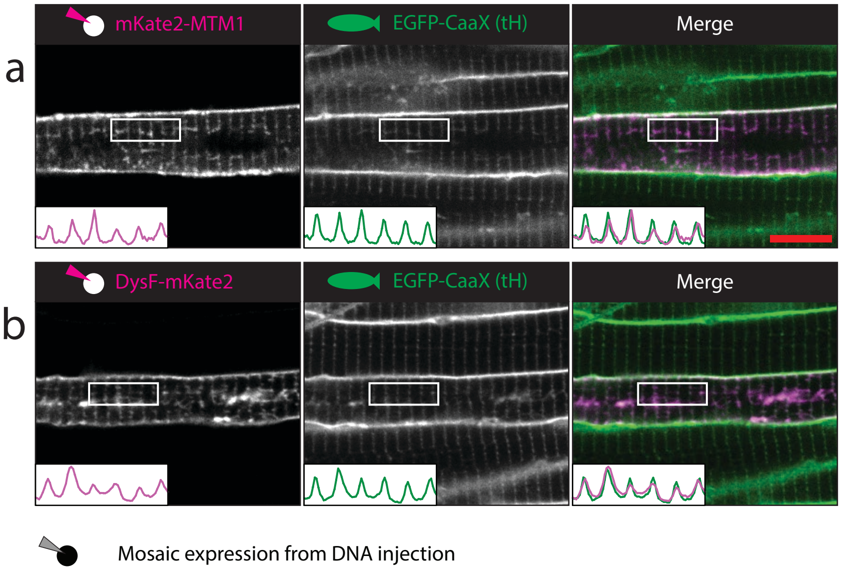

Myotubularin and Dysferlin mark the early, CaaX-positive T-tubules.
Mosaic transgenic expression of T-tubule markers verifies that the CaaX-positive, transverse, intracellular membranes are T-tubules. (a) Myotubularin. (b) Dysferlin. Scale bar, 10µm. Images are representative of 12 individual cells within different individual animals. Related to Figure 1

Supplementary Figure 2

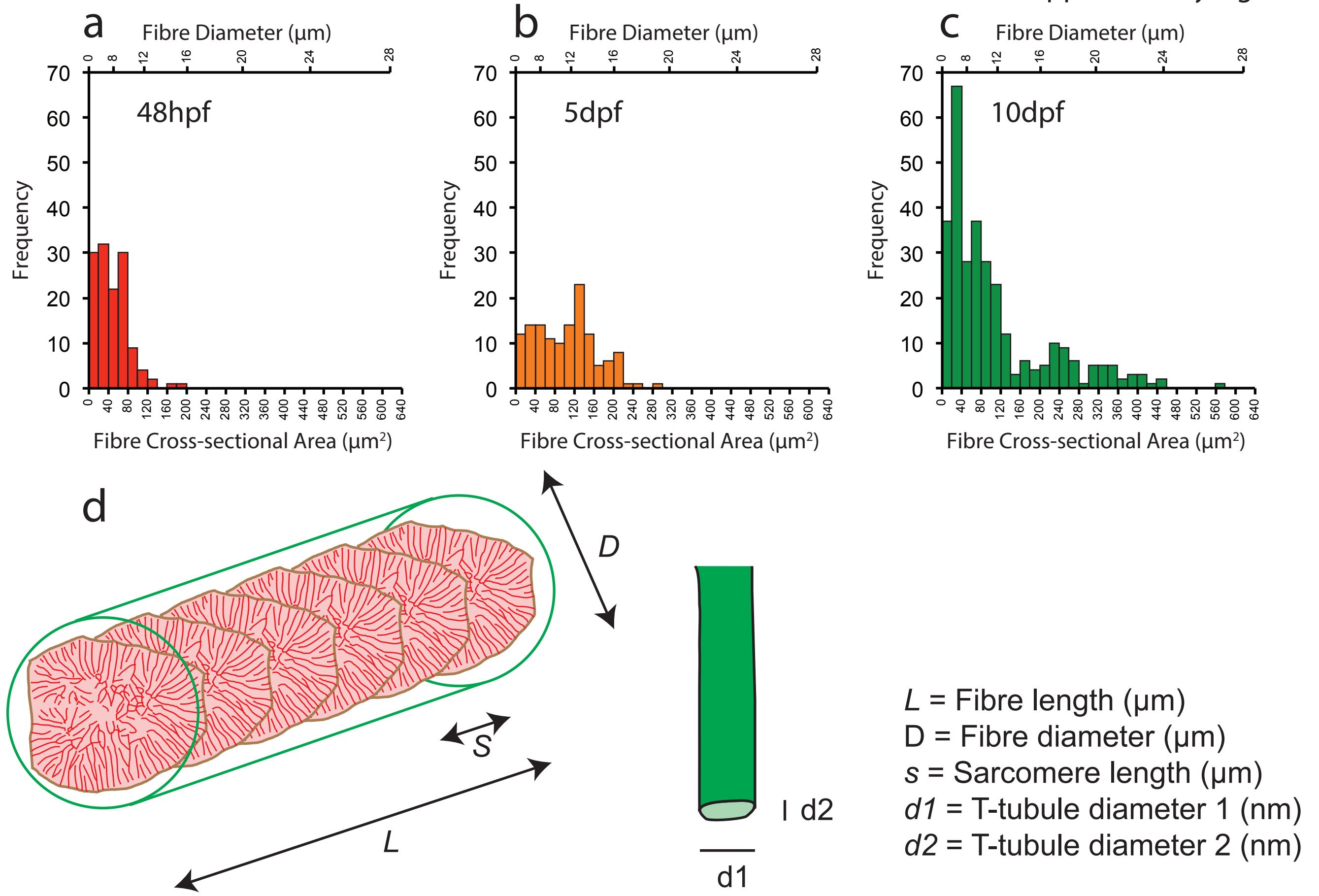

e

$$\frac{1}{1000}\times\pi\left(3\left[\frac{d1}{2}+\frac{d2}{2}\right]-\sqrt{\left[3\cdot\frac{d1}{2}+\frac{d2}{2}\right]\left[\frac{d1}{2}+3\cdot\frac{d2}{2}\right]}\right)\times\frac{L}{s}\times 1.26\left[0.95\left(\pi\left(\frac{D}{2}\right)^2\right)-13.55\right]+6.06$$

Conversion from nm to µm for tubule perimeter

Perimeter of T-tubule cross-sectional ellipse (Ramanujan's approximation)

Number of sarcomeric units (fibre length/sarcomere length)

Total linear furrow distance per sarcomere (TT length; see alse Fig. 3e)

Myofibril cross sectional area (see also Fig. 3d)

Fibre cross sectional area

f

$$\frac{3L\pi(3d1+3d2-\sqrt{(3d1+d2)(d1+3d2)})(399\pi D^2-14684)}{8000000s}$$

g

$$\frac{157.939995L\pi(1.197\pi D^2)-44.052}{15200}$$

A mathematical model for T-tubule formation
We used empirical data to produce a mathematical model of the surface area of membrane present in the T-tubule system of muscle fibres of any given length and diameter. (a-c) Muscle fibre cellularity profiles at (a), 48hpf (b) 5dpf (c) 10dpf based on directly measured cross-sectional areas (bottom axis). The top axis represents the equivalent diameter of a perfect circle with the same cross-sectional area. (d) Schematic of a model muscle fibre illustrating the parameters used to calculate T-tubule and sarcolemmal surface area. (e) Equation for T-tubule surface area based on the input parameters shown in d. Sections of the equation are colour coded to show source. (f) Simplified version of the formula shown in e, with the same input parameters. (g) Simplified version of the equations shown in e and f, with the constant parameters d1, d2 and s amalgamated, leaving only L and D. A third version of the equation where diameter (D) is replaced by cross sectional area is also given in the Methods. Related to Figure 3

Supplementary Figure 3

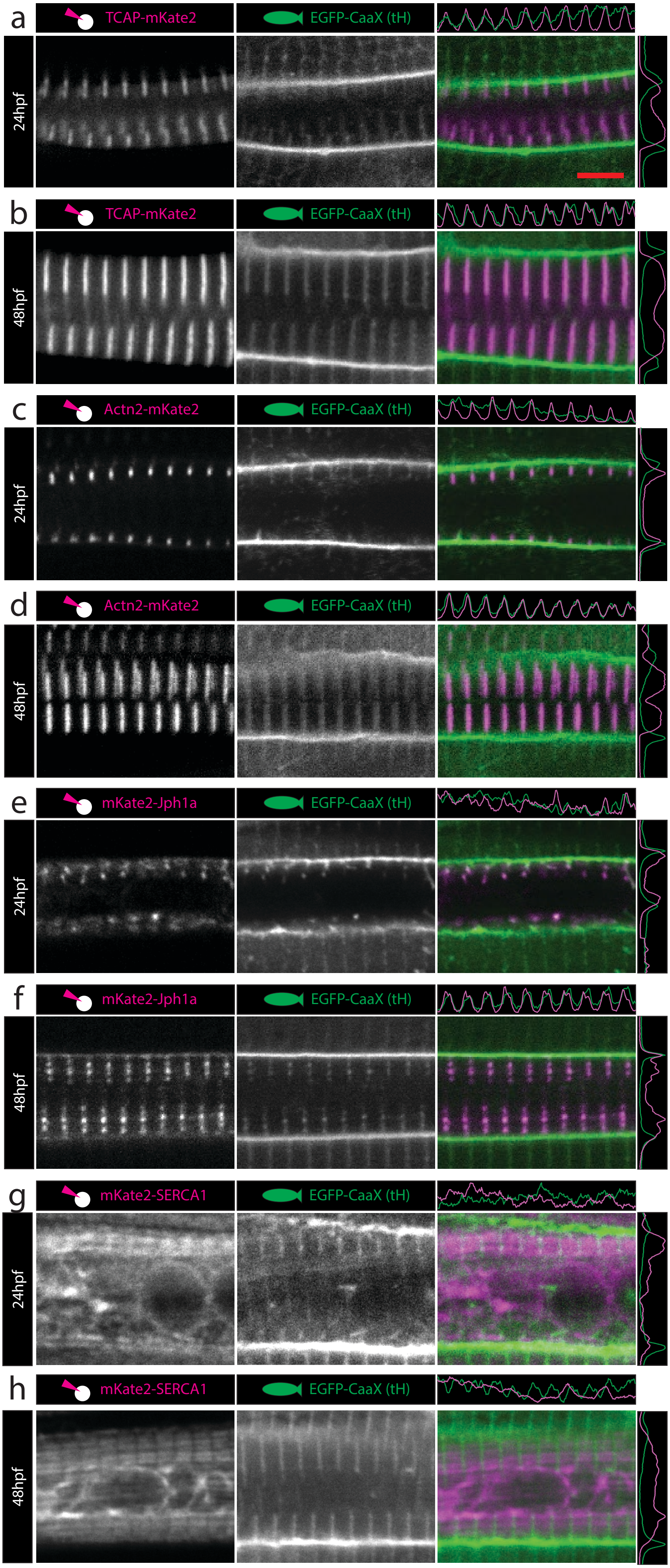

Markers of sarcomere formation and organized sarcoplasmic reticulum are associated spatially with forming T-tubules. Mosaic transgenic expression of sarcomere and sarcoplasmic reticulum markers shows a tight association with the forming T-tubules. (a, b) Titin cap (TCAP/telethonin). (c, d) Alpha-actinin2 (actn2). (e, f) Junctophilin 1a (Jph1a). (g-h) sarcoplasmic/endoplasmic reticulum calcium ATPase 1 (SERCA1). (a, c, e, g) 24hpf; (b, d, f, h), 48hpf. Scale bar, 5μm. Images are representative of 12 individual cells within different individual animals. Related to Figure 4.

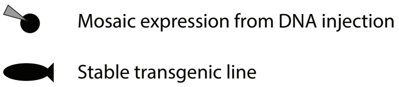

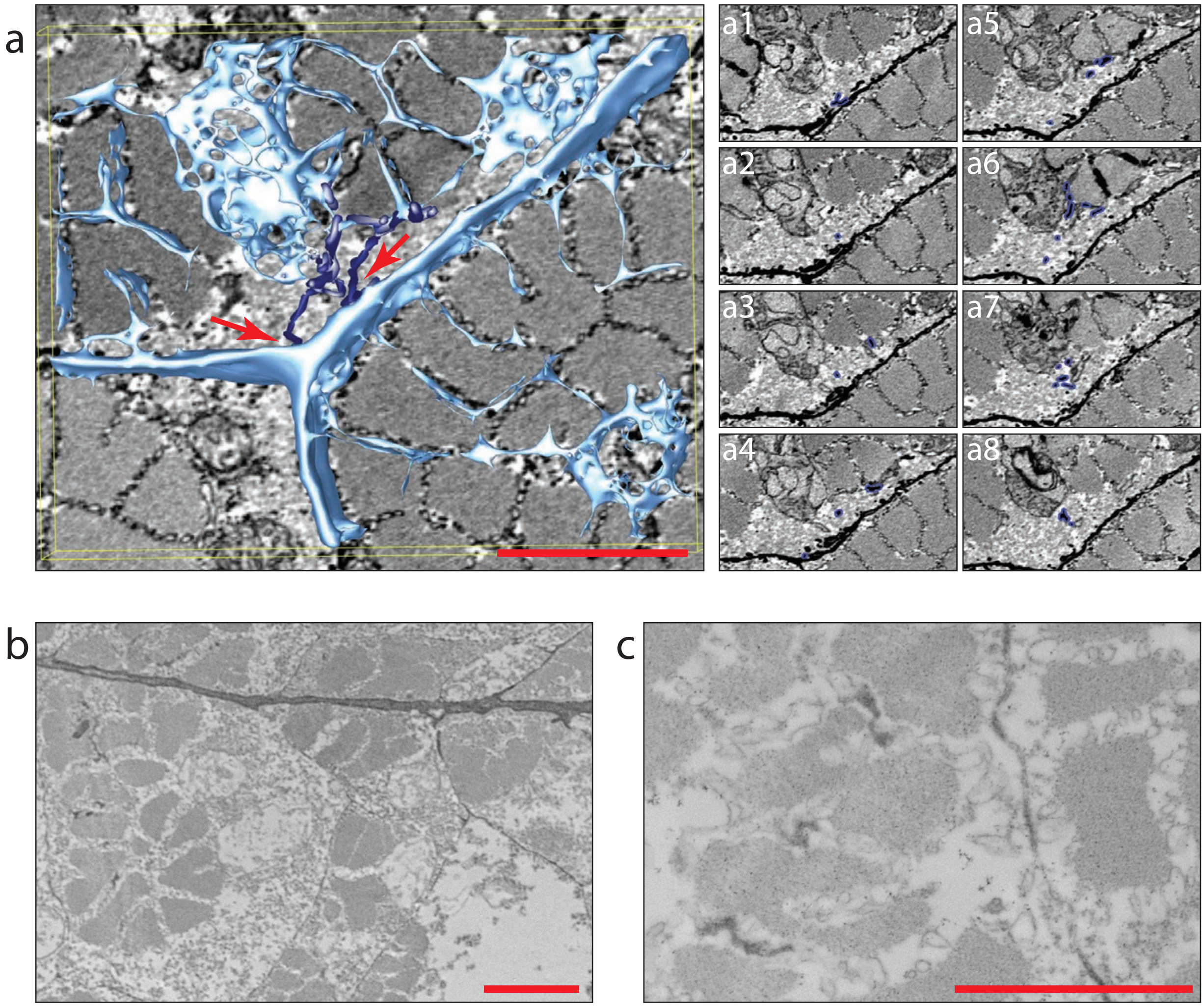

Surface connected tubules are stabilised by endocytic capture

(a) Serial blockface electron microscopy and 3D reconstruction shows tubules connecting stabilized, sarcomere associated tubules to the sarcolemma. Where the sarcomere is some distance from the sarcolemma, connecting tubules are often unstructured and looping (arrows). Individual Z planes are shown in a1-a8. (b, c) Injection of unconjugated horse radish peroxidase into the circulation at 48hpf results in infiltration into the T-tubules after 45 min, indicated by a relatively high electron density. Scale bars, 5μm. All images were derived from two individual animals and images shown are representative of both. Related to Figure 5.

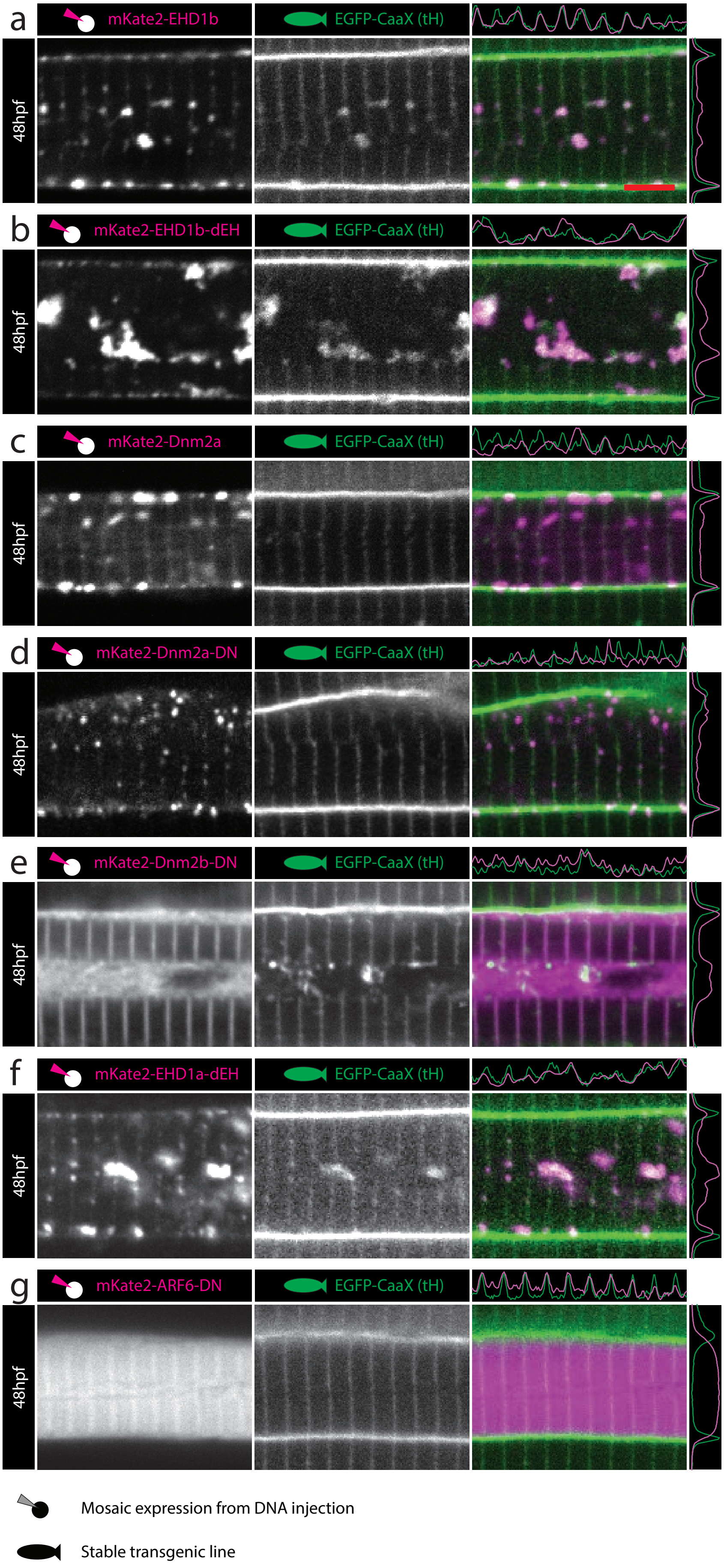

Selected endocytic markers localize to the T-tubules and dominant negative forms perturb T-tubule formation. Mosaic transgenic expression of certain endocytic proteins shows association with the forming T-tubules. When dominant negative forms are expressed the result is often perturbing.
(a) EHD1b (b) EHD1b-dEH (c) Dnm2a (d) Dnm2a-DN (e) Dnm2b-DN (compare with Fig. 6g) (f) EHD1a-dEH (compare with Fig. 6d). (g) ARF6-DN (compare with Fig. 6h). Scale bar, 5μm. Images are representative of 12 individual cells within different individual animals. Related to Figure 6.

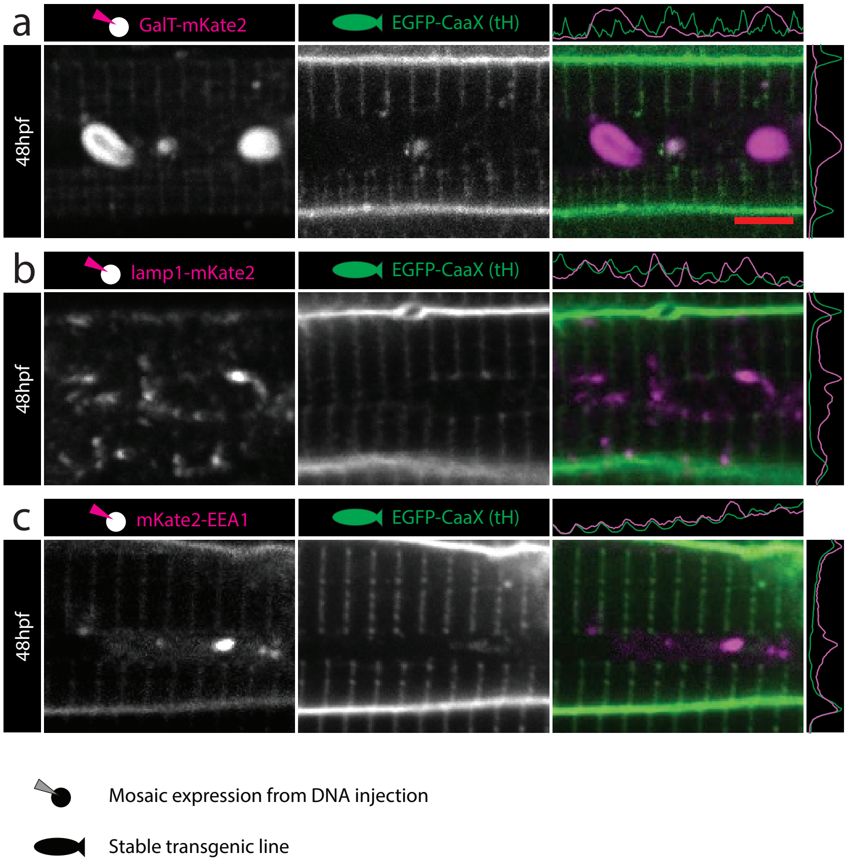

Non-phosphoinositide binding markers of intracellular domains.
Mosaic transgenic expression of proteins specific for selected intracellular domains shows comparable expression patterns to phosphoinositide markers. (a) 1,4-galactosyltransferase (GalT) marks the Golgi complex (compare with FAPP1, Fig. 7e). (b) Lysosomal associated membrane protein 1 (LAMP1) marks the lysosomal compartment (compare with ATG18, Fig. 7f). (c) Early endosome associated 1 (EEA) marks the early endosomal compartment (compare with 2xFYVEhrs, Fig. 7g). Scale bars, 5µm. Images are representative of 12 individual cells within different individual animals. Related to Figure 7.

Supplementary Figure 7

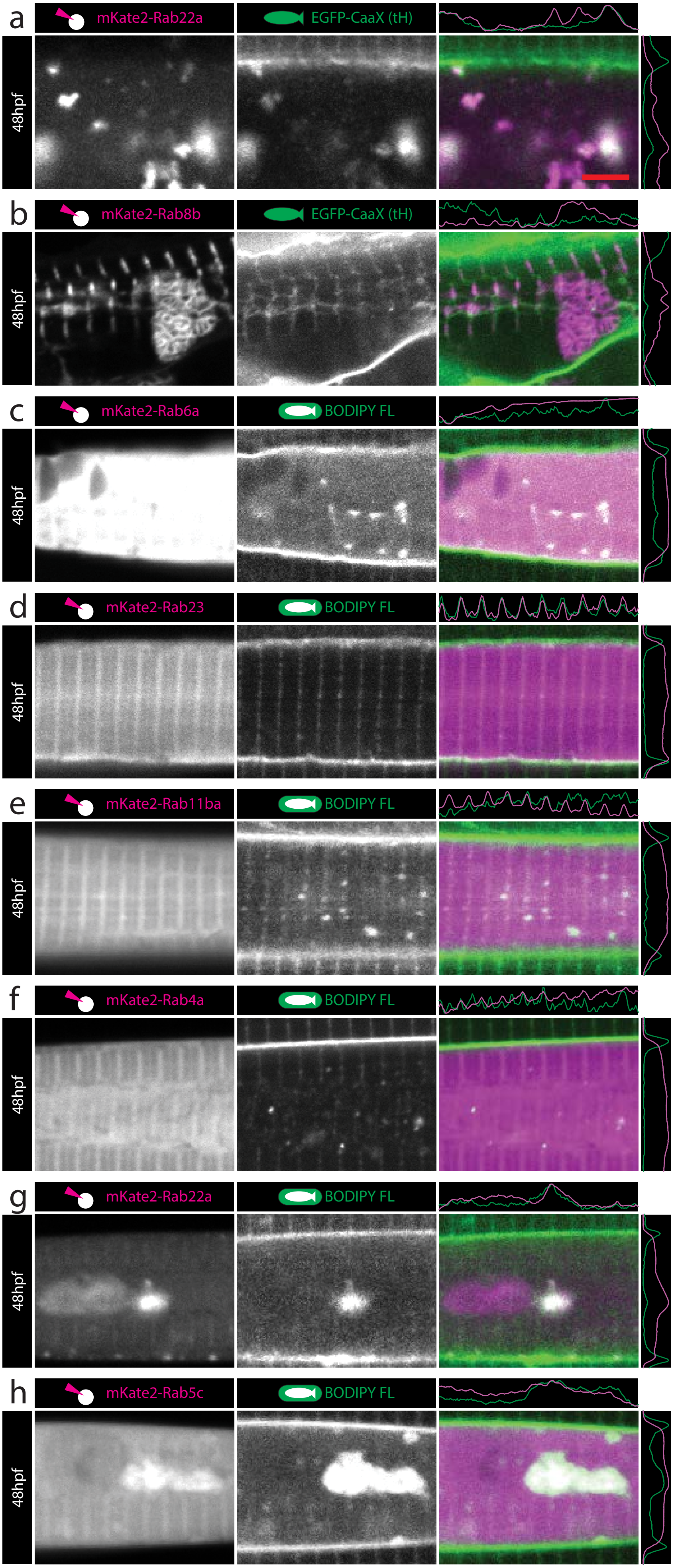

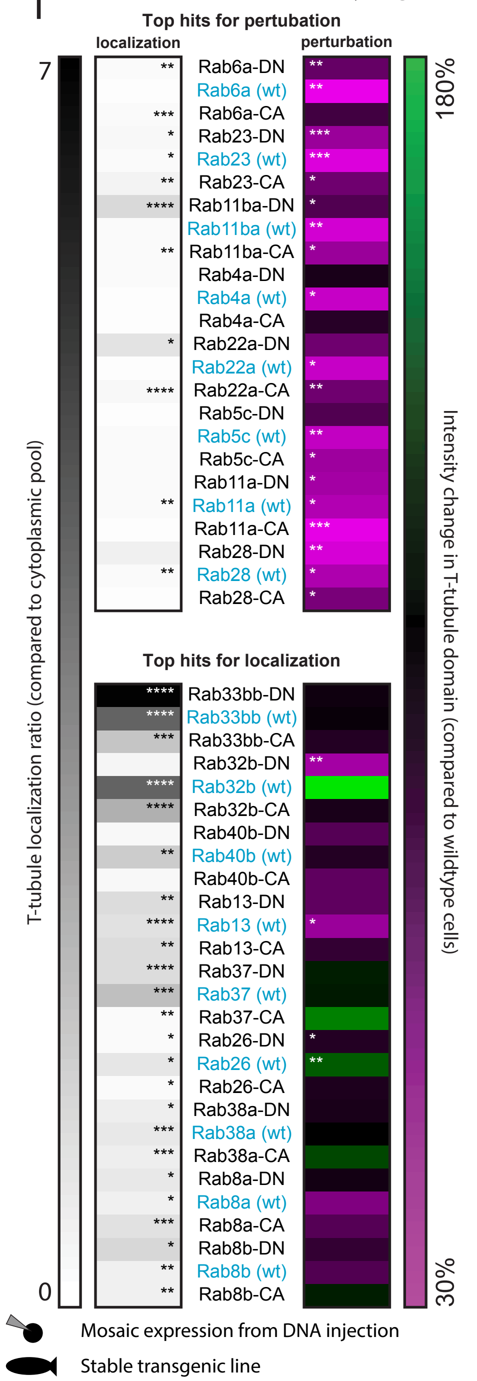

Expression screen of Rabs identifies proteins with specific localization, and which can perturb T-tubule formation. (a) Rab22a. (b) Rab8b ectopic tubular knots. (c-h) BODIPY-FLC5 staining of top hits. (c) Rab6a. (d) Rab23. (e) Rab11ba. (f) Rab4a. (g) Rab22a. (h) Rab5c. (i) Quantitative analysis using dominant negative and constitutively active Rabs. n = 6 fibres from individual independent animals per condition, measured over one independent experiment. Two-tailed T-test. *$p < 0.05$, **$p < 0.01$, ***$p < 0.001$, ****$p < 0.0001$. Exact p values are given in source data. Scale bars, 5µm. Images are representative of 12 individual cells within different animals. Related to Figure 8

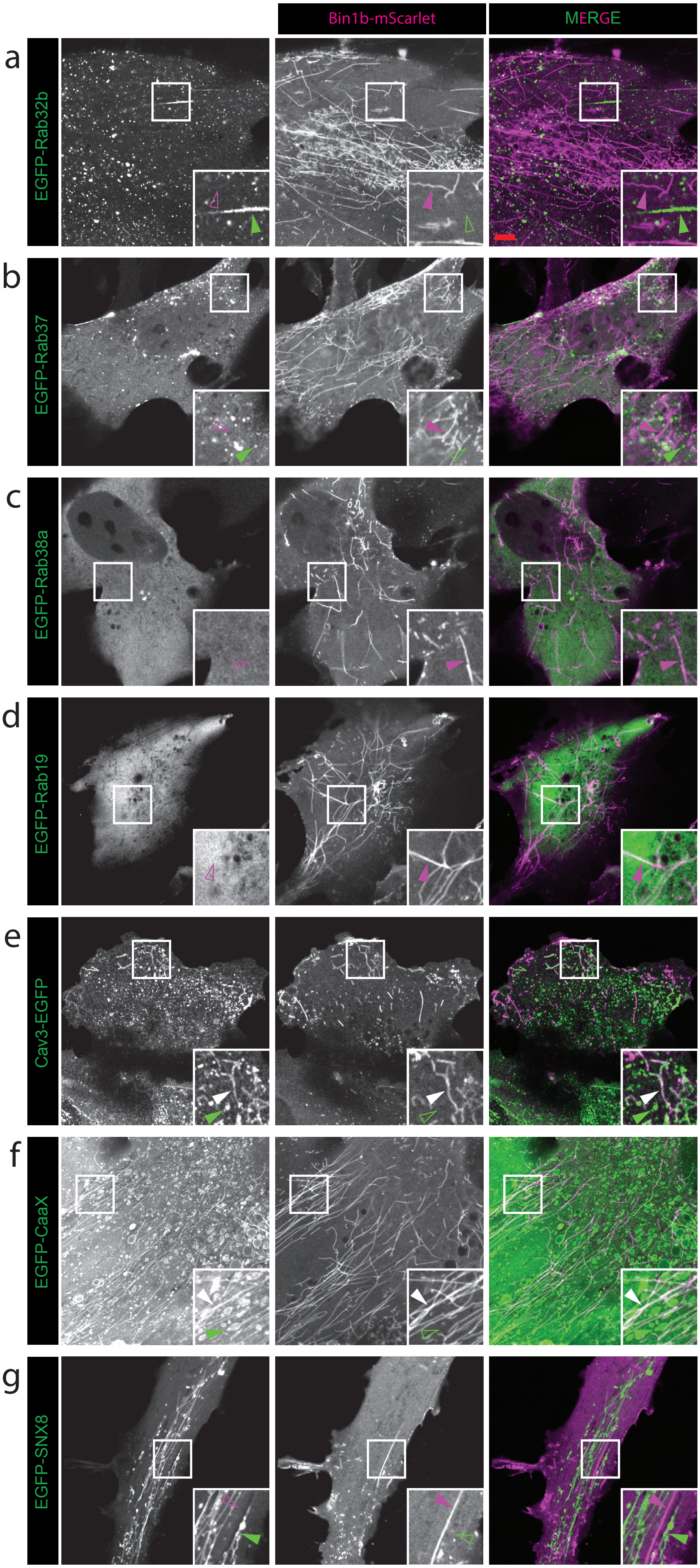

An *in vitro* model of T-tubule formation for validation of factors involved in T-tubule development. BHK cells are transfected with zebrafish Bin1b-mScarlet to induce tubule formation. Selected EGFP-tagged proteins are co-transfected in order to test their association with the Bin1b induced tubules. (a) Rab32b. (b) Rab37. (c) Rab38a. (d) Rab19. (e) Caveolin3. (f) CaaX. (g) Snx8. Images are representative of three individual cells imaged within the same experiment. Related to Figure 9.

	Reference	Allele number
bact2-Lifeact-mKate2	This study	uq3rp
bact2-mKate2-KDEL	This study	uq4rp
Slow myosin heavy chain 1 knockout	This study	uq20rp
Titin.1 knockout	This study	uq21rp
acta1-EGFP-CaaX (Hras)	1	pc10

Supplementary Table 1. Transgenic and mutant zebrafish lines used in this study

Macro or Algorithm	Reference
GEN_Split_to_tif_CZI_AND_LSM.ijm (1)	This study
GEN_Morphometrics.ijm (2)	This study
GEN_Split_to_green_and_blue_highlight_saturated.ijm (3)	This study
TT_GET_box_ROIs_for_amplitude_comparison.ijm (4)	This study
TT_perturbation_process.ijm (5)	This study
TT_GET_circle_ROIs_for_localisation_comparison.ijm (6)	This study
GEN_Put_circle.ijm (7)	This study
TT_localisation_comparison_process.ijm (8)	This study
Export ROIs in RoiManager to a SVG file (Python script)	BioImage Analysis Wiki

Supplementary Table 2. Software and algorithms used in this study

Supplementary Reference

1. Williams, R.J. *et al.* The *in vivo* performance of an enzyme-assisted self-assembled peptide/protein hydrogel. *Biomaterials* **32**, 5304-5310 (2011).
