# Supplementary material for: In vivo cell biological screening identifies an endocytic capture mechanism for T-tubule formation
Source: Nat Commun. 2020 Jul 24;11:3711. doi: 10.1038/s41467-020-17486-w (PMC7381618; doi:10.1038/s41467-020-17486-w)
Supplement: Supplementary file 8 — Description of Additional Supplementary Files [file 41467_2020_17486_MOESM8_ESM.pdf]

**Title:** Supplementary Movie 1
**Description:** Confocal timelapse series showing initial formation of the skeletal muscle T-tubule system from 18hpf to 31hpf. At 18hpf unstructured tubules can be seen adjacent to the sarcolemma (red arrows). The first tubules adopting regular, reiterated spacing are marked by blue arrows. Movies are representative of four individual animals imaged within the same experiment.

**Title:** Supplementary Movie 2
**Description:** Confocal timelapse series showing initial formation of the skeletal muscle T-tubule system from 30hpf to 39hpf. Transient longitudinal elements can be seen between transverse tubules (blue arrows). Movies are representative of four individual animals imaged within the same experiment.

**Title:** Supplementary Movie 3
**Description:** Serial blockface scanning electron microscope volume in transverse orientation at 48hpf. The sequence pans through serial sections showing heavily stained T-tubules at the Z-lines. It then returns back through the volume displaying a thresholded surface which distinguishes T-tubules, sarcolemma and mitochondria. Movies are representative of two individual animals imaged within the same experiment.

**Title:** Supplementary Movie 4
**Description:** Confocal timelapse series showing a lateral view of a double transgenic zebrafish expressing Lifeact-mKate2 (magenta) and EGFP-CaaX (green). As the actin-rich myofibrils develop, T-tubules form at regular intervals along their length. Structured tubules are invariably associated with myofibrils (blue arrows). Movies are representative of four individual animals imaged within the same experiment.

**Title:** Supplementary Movie 5
**Description:** 24hpf EGFP-CaaX zebrafish injected with the low molecular weight tracer Alexa-647-UTP (magenta). At the start of the sequence, tracer is already present on the sarcolemma and in the T-tubules. After several minutes, tracer can be seen in intracellular vesicles (blue arrows). Movies are representative of three individual animals imaged within the same experiment.

**Title:** Supplementary Data 1
**Description:** Full list of plasmids used in this study and their unique repository identifiers.

**Title:** Supplementary Software
**Description:** Custom Fiji (ImageJ) macros files used in this study (see also Supplementary Table 2).
