# Supplementary material for: In vivo cell biological screening identifies an endocytic capture mechanism for T-tubule formation
Source: Nat Commun. 2020 Jul 24;11:3711. doi: 10.1038/s41467-020-17486-w (PMC7381618; doi:10.1038/s41467-020-17486-w)
Supplement: Supplementary file 11 — Reporting Summary [file 41467_2020_17486_MOESM11_ESM.pdf]

Corresponding author(s): Robert Parton/Thomas Hall

Last updated by author(s): May 26, 2020

Reporting Summary

Nature Research wishes to improve the reproducibility of the work that we publish. This form provides structure for consistency and transparency in reporting. For further information on Nature Research policies, see our Editorial Policies and the Editorial Policy Checklist.

Statistics

For all statistical analyses, confirm that the following items are present in the figure legend, table legend, main text, or Methods section.

n/a	Confirmed	
☐	☒	The exact sample size (n) for each experimental group/condition, given as a discrete number and unit of measurement
☒	☐	A statement on whether measurements were taken from distinct samples or whether the same sample was measured repeatedly
☐	☒	The statistical test(s) used AND whether they are one- or two-sided<br>*Only common tests should be described solely by name; describe more complex techniques in the Methods section.*
☒	☐	A description of all covariates tested
☐	☒	A description of any assumptions or corrections, such as tests of normality and adjustment for multiple comparisons
☐	☒	A full description of the statistical parameters including central tendency (e.g. means) or other basic estimates (e.g. regression coefficient) AND variation (e.g. standard deviation) or associated estimates of uncertainty (e.g. confidence intervals)
☐	☒	For null hypothesis testing, the test statistic (e.g. F, t, r) with confidence intervals, effect sizes, degrees of freedom and P value noted<br>*Give P values as exact values whenever suitable.*
☒	☐	For Bayesian analysis, information on the choice of priors and Markov chain Monte Carlo settings
☒	☐	For hierarchical and complex designs, identification of the appropriate level for tests and full reporting of outcomes
☒	☐	Estimates of effect sizes (e.g. Cohen's d, Pearson's r), indicating how they were calculated

Our web collection on statistics for biologists contains articles on many of the points above.

Software and code

Policy information about availability of computer code

Data collection	Commercial software: Zeiss Zen 2012 Black (confocal microscopy). Olympus iTEM 5.2. Custom code provided in "Supplementary Macro Code": GEN_Morphometrics.ijm (Macro2), TT_GET_box_ROIs_for_amplitude_comparison.ijm (Macro 4), TT_GET_circle_ROIs_for_localisation_comparison.ijm (Macro 6), GEN_Put_circle.ijm (Macro 7).
Data analysis	Commercial software: Adobe Photoshop CC 2019. Adobe Illustrator CC 2019. IMOD 4.9 (electron microscopy), Microsoft Excel for Mac v14.7.1, Fiji (ImageJ) v2.0.0-rc-69/1.52t, ROI to .svg (python script) Bioimage Analysis Wiki, GraphPad Prism 8.2.0. Custom code provided in "Supplementary Macro Code": GEN_Split_to_tif_CZI_AND_LSM.ijm (Macro1), GEN_Split_to_green_and_blue_highlight_saturated.ijm (Macro 3), TT_perturbation_process.ijm (Macro 5), TT_localisation_comparison_process.ijm (Macro 8)

For manuscripts utilizing custom algorithms or software that are central to the research but not yet described in published literature, software must be made available to editors and reviewers. We strongly encourage code deposition in a community repository (e.g. GitHub). See the Nature Research guidelines for submitting code & software for further information.

Data

Policy information about availability of data

All manuscripts must include a data availability statement. This statement should provide the following information, where applicable:

- Accession codes, unique identifiers, or web links for publicly available datasets
- A list of figures that have associated raw data
- A description of any restrictions on data availability

Data from the zebrafish Bio-Atlas was used in this study as described (http://bio-atlas.psu.edu/).
All plasmids created and used in this study have been made available from a not for profit repository (Addgene; https://www.addgene.org/Rob_Parton/). A full list along with unique identifiers are given in Supplementary Table 1.

The source data underlying Figs 3d, 3e, 3f, 3g, 4g, 4k, 6j, 6m, 7h, 8i and Supplementary Figs 3a, 3b, 3c, 8i are provided as a Source Data file.

Field-specific reporting

Please select the one below that is the best fit for your research. If you are not sure, read the appropriate sections before making your selection.

☒ Life sciences ☐ Behavioural & social sciences ☐ Ecological, evolutionary & environmental sciences

For a reference copy of the document with all sections, see nature.com/documents/nr-reporting-summary-flat.pdf

Life sciences study design

All studies must disclose on these points even when the disclosure is negative.

Sample size	For the non-quantitative data in Figure 1a-f, Figure 4a-f, Supplementary Figure 4a-h, Figure 5a-c, Figure 7e-g, Supplementary Figure 7a-c, 12 individual animals were observed. No sample size calculation was carried out because no qualitative differences in expression pattern between replicates were detected. TEM images in figures 2a-f and were derived from 2 individual animals per time point and specific observations described were noted in >12 individual cells. Serial blockface electron microscopy in Figure 2g-h, Figure 5h and Supplementary Figure 5a was carried out on 2 animals and the specific observations described were noted in both individuals. The focussed ion beam electron microscope images in Figure 2j were taken from a single individual animal, as were the transmission electron microscopy montages shown in figure 3a-c. No sample size calculations were carried out. Sample sizes for the EM represent the maximum we could achieve given the technically demanding and labour intensive nature of the experiments. Similarly, TEM images in Figures 4h-j were derived from 2 individual animals per condition. Differences between the three conditions measured were significant to p <0.0001. The quantitative analyses shown in Figures 6, 7, 8 and Supplementary Figures 6 and 8 were carried out on 6 muscle fibres per condition. No sample size calculation was carried out. However in each case a pilot study was carried out to qualitatively assess variation in the expression pattern of each construct in >6 cells per construct and to gain information on which compartment was labelled. In these analyses a number of markers are enriched on the T-tubule domain with significance values to p <0.0001 indicating sufficient power in the analyses. Since this was primarily a medium throughput screening approach 6 cells per construct was the maximum we were able to achieve. For the colocalisation experiments shown in Figure 9a-f and supplementary Figure 9a-g, three samples per condition were imaged. The extent of colocalisation between samples from each condition was invariant, and the summary graphic in Figure 9g reflects all three samples imaged in each case.
Data exclusions	There were no data exclusions in this study
Replication	For the non-quantitative data in Figures 1a-f, 4a-f, 5a-c, 7e-g and Supplementary Figures 4a-h, 7a-c, and for the quantitative data in Figures Figures 6, 7, 8 and Supplementary Figures 6 and 8, pilot studies were carried out to assess which compartment markers localized to and the extent of variation between samples from the same condition. All subsequent quantitative and non-quantitative observations were consistent with these initial pilot studies. As such, these experiments were performed independently twice (although direct quantitation was only performed once). The dextran/Alexa-647 injection experiments in Figures 5d-g were replicated independently three times. Electron microscopy was was performed on a minimum of 2 animals per experimental condition but independent replication was not carried out due to the technically demanding and labour intensive nature of the experiments.
Randomization	No randomization was used to determine sample allocation. For cellular phenotyping experiments, affected cells were compared to non affected within the same animal (internal control). For experiments with CRISPR mutants, control and mutant animals came from the same clutches of heterozygote incrossed animals which were sorted for homozygosity based on phenotype. They were processed and scored at the same time.
Blinding	Blinding was not possible in these experiments, since cells had to be picked based on whether or not they were expressing a transgene. SImilarly, mutant and control fish from the same clutch were picked based on phenotype.

Reporting for specific materials, systems and methods

We require information from authors about some types of materials, experimental systems and methods used in many studies. Here, indicate whether each material, system or method listed is relevant to your study. If you are not sure if a list item applies to your research, read the appropriate section before selecting a response.

Materials & experimental systems

n/a	Involved in the study	
☒	☐	Antibodies
☐	☒	Eukaryotic cell lines
☒	☐	Palaeontology and archaeology
☐	☒	Animals and other organisms
☒	☐	Human research participants
☒	☐	Clinical data
☒	☐	Dual use research of concern

Methods

n/a	Involved in the study	
☒	☐	ChIP-seq
☒	☐	Flow cytometry
☒	☐	MRI-based neuroimaging

Eukaryotic cell lines

Policy information about cell lines

Cell line source(s)	BHK-21[C-13]. ATCC-CCL-10. All cells used were between passage 9 and 13
Authentication	This cell line was not authenticated but only early passages were used from cells supplied from ATCC
Mycoplasma contamination	All cell lines in use in our laboratory are subject to a quarterly mycoplasma testing regime using the Lonza MycoAlert Mycoplasma detection kit (LT07-418)
Commonly misidentified lines (See ICLAC register)	*Name any commonly misidentified cell lines used in the study and provide a rationale for their use.*

Animals and other organisms

Policy information about studies involving animals; ARRIVE guidelines recommended for reporting animal research

Laboratory animals	Zebrafish (Danio rerio) of indeterminate sex, between 3 and 10 days post fertilization depending on experiment
Wild animals	*Provide details on animals observed in or captured in the field; report species, sex and age where possible. Describe how animals were caught and transported and what happened to captive animals after the study (if killed, explain why and describe method; if released, say where and when) OR state that the study did not involve wild animals.*
Field-collected samples	*For laboratory work with field-collected samples, describe all relevant parameters such as housing, maintenance, temperature, photoperiod and end-of-experiment protocol OR state that the study did not involve samples collected from the field.*
Ethics oversight	All experimental procedures were reviewed and approved by the Molecular Biosciences Animal Ethics Committee at the University of Queensland prior to commencement.

Note that full information on the approval of the study protocol must also be provided in the manuscript.
